# Identification of Wb123 as an Early and Specific Marker of *Wuchereria bancrofti* Infection

**DOI:** 10.1371/journal.pntd.0001930

**Published:** 2012-12-06

**Authors:** Joseph Kubofcik, Doran L. Fink, Thomas B. Nutman

**Affiliations:** Laboratory of Parasitic Diseases, National Institute of Allergy and Infectious Diseases, National Institutes of Health, Bethesda, Maryland, United States of America; Michigan State University, United States of America

## Abstract

**Background:**

The current antibody tests used for monitoring in lymphatic filariasis (LF) elimination programs suffer from poor specificity because of the considerable geographical overlap with other filarial infections such as *Loa loa (*Ll*)*, *Onchocerca volvulus (*Ov*)*, and *Mansonella* perstans (Mp).

**Methods:**

Using bioinformatics to assemble into contigs 2048 expressed sequence tags (ESTs) from the L3 infective larvae of *W. bancrofti* (Wb), these were next assessed for homology to known proteins and nucleotides and to similar assemblies of L3 larval ESTs of *B. malayi* (Bm – n = 5068), Ov (n* = *4166), and Ll (n* = *3315). Nineteen potential L3- and Wb- and/or Bm-specific antigens were identified. Sixteen of the 19 antigens could be expressed as fusion proteins with Renilla luciferase (Ruc); these were used in a rapid Luciferase Immunopreciptation System (LIPS) assay.

**Results:**

One of the 16 expressed antigens (Wb123) was both highly immunogenic and specific for Wb. Using Wb123-based IgG and IgG4 LIPS assays on well-defined sera from normal North Americans and those infected exclusively with intestinal helminths, we could detect all of the Wb-infected individuals (from diverse geographic regions) with 100% sensitivity and 100% specificity. Using sera from exclusively Ll-infected, Ov-infected Mp-infected or Bm-infected subjects as the negative comparator, the sensitivities were between 98–100% and the specificities ranged between 84–100% (for IgG anti-Wb123) and between 98–100% (for IgG4 anti-Wb123). Blinded assessments using panels of sera from various Wb-, Bm- or non-Wb helminth-infected subjects demonstrated equally high degrees of sensitivity and specificity.

**Significance:**

We have identified a Wb-encoded antigen that can be used both as a rapid, high throughput tool to diagnose individual *Wb* infections and as a sensitive method for early detection of recrudescent infections in areas of control and for mapping new areas of Wb transmission.

## Author Summary

To address an unmet need for a surveillance tool to detect transmission of *Wuchereria bancrofti* (Wb), a causative agent of lymphatic filariasis, following mass drug administration (MDA) control measures, we used a novel bioinformatic approach to identify Wb-specific antigens expressed primarily by the infective stage larvae of Wb that could be used as the basis for a rapid, high throughput antibody assay. From 19 potential candidates, we identified one, termed Wb123, that could be used as the basis for the rapid detection of IgG and IgG4 reactivity in a luciferase immunoprecipitation system (LIPS) assay. These anti-Wb123 IgG and IgG4 antibodies were only present in those with Wb infection and not in those infected with closely related filarial parasites (e.g. *Onchocerca volvulus*, *Loa loa*, *Mansonella perstans*) that share the same geographical niches with Wb. Our data suggest that this rapid Wb123 assay has the potential to be used in for large scale detection of recrudescent Wb infection and thereby may provide a new tool for the monitoring of transmission control measures.

## Introduction

As one of the neglected tropical diseases (NTDs), lymphatic filariasis (LF) caused by the filarial parasites *Wuchereria bancrofti* (Wb), *Brugia malayi* (Bm) and *Brugia timori* can lead to disfiguring and disabling lymphedema and elephantiasis. Past and ongoing control measures, aimed at interrupting transmission by eliminating the reservoir of infection (through mass drug administration [MDA] wherever possible, e.g., Global Program for the Elimination of Lymphatic Filariasis [GPELF]) has led to substantial decreases in the prevalence of infection and the risk of disease [1]. Despite these measures, estimates suggest that 120 million people remain infected with Wb or Bm with an additional 1 billion people being at risk in the tropics and subtropics worldwide [2]. Superimposed on this estimate of Wb- and Bm-infected individuals is the concern about the serious adverse events associated with MDA in West and Central Africa in areas where another filarial parasite, *Loa loa* [Ll], is co-endemic [3], [4].

MDA programs are in progress in more than 50 of the 72 LF-endemic countries, with 13 having stopped following at least 5 annual MDA treatments [1]. Currently, WHO guidelines for assessment of transmission interruption are based on the monitoring of antigenemia (for Wb at least) in children; however, antibody responses – particularly to antigens expressed in L3s (the infective larvae) are likely to provide much earlier measures of ongoing transmission [5], [6] than the presence of microfilariae or circulating filarial antigen. Such an approach has been used quite successfully in onchocerciasis-endemic regions of Central America where the absence of antibody responses to an *O. volvulus* (Ov) L3-expressed antigen, Ov16 [7], has been used as one of the criteria to certify areas free of Ov transmission [8]–[11].

A number of immunoassays using a variety of different filarial Ags have been proposed for use as surveillance tools in LF; these include Bm14/BmSXP-1 [12], BmR1 [13], WbSXP-1 [14], and Bm33 [15]. The sensitivity of these assays has generally been high but limited specificity with respect to non-LF causing filariae (Ov, Ll, *Mansonella spp.*) often co-endemic with Wb in Africa and parts of the Americas will likely limit their utility in these regions.

In the present study, we utilized a bioinformatics approach to identify potential antigens that are expressed in post-parasitic L3s, that would be Wb- (and/or Bm-specific), and that in turn could be used in a rapid, high throughput format to provide a sensitive method for early detection of recrudescent infections in areas of control, for mapping new areas of transmission of Wb infection, and potentially for certifying regions/countries as free of transmission.

## Methods

### Ethics statement

All samples used were acquired under a number of registered protocols that were approved by the Institutional Review Board of NIAID with the majority being collected under either NCT00001230, NCT00342576. Written informed consent was obtained from all subjects prior to collection of the samples.

#### Contig construction and bioinformatics analysis

*Brugia malayi* L3 ESTs and *Wuchereria bancrofti* L3 ESTs (downloaded as FASTA files from Genbank, NCBI, NLM) were assembled into contigs using the Desktop cDNA Annotation System (dCAS 1.4.3) software package [16]. The resulting output, and Excel table with hyperlinks, was used to identify potential proteins that were specific for the lymphatic filariae *Wuchereria bancrofti* (Wb) and/or *Brugia malayi* (Bm) and that were without significant homology to the related filariae (*Loa loa* [Ll] and *Onchocerca volvulus* [Ov]). Contigs were selected for further evaluation as candidate assay targets based on: 1) length of at least 200 bp with a predicted open reading frame (ORF); and 2) lack of sequence homology to the non-redundant protein database (nr) and other stages (mf, adult males, adult females) of Bm or Wb (Table S1).

### Plasmid and primers

Each of the 19 potential targets (full length or longest assembled contig) was synthesized commercially (Genscript, Piscataway, NJ) with codon usage optimized for expression in mammalian cells. Using insert specific primers containing BamH1 and Xho1 modifications, each of these 19 DNA inserts was amplified and cloned into the BamH1/Xho1 site of pREN2, a mammalian Ruc expression vector described previously [17]. The resulting pREN2 expression vector was prepared using a Qiagen Midi kit (Qiagen, Gaithersburg, MD). Automated DNA sequencing was used to confirm the integrity of the DNA constructs. Ruc-antigen fusion extracts were prepared from transfected COS1 cells as previously described [17].

### Serum samples

Serum samples (Table 1 and Table S2) from patients with filarial infections were chosen in such a way that only a single infecting species of filariae was present either because of geographic constraints and/or by definitive identification of a particular parasite and exclusion of LF (circulating antigen negative by TropBio ELISA). For the blinded analyses, well-characterized de-identified serum collections were used (kindly provided by Drs. Patrick Lammie and Shenoy).

**Table 1:** Samples used to assess sensitivity and specificity of Wb123 LIPS.

Group	Country source of population	Number of samples
Normal unexposed		
	Canada	2
	United States	50
	Total	52
*Wuchereria bancrofti*		
	Cook Islands (Mf+)	6
	Comoros Islands (Mf+)	1
	India (Mf+)	27
	India (Mf-Cag+)	7
	Guyana (Mf+)	2
	Total	43
*Onchocerca volvulus* (mf+)		
	Ecuador	24
	Guatemala	10
	Sierra Leone	2
	Cameroon*	2
	Total	38
*Loa loa* (mf+)		
	Benin	55
	Cameroon	3
	Gabon	2
	Central African Republic	2
	Total	62
*Mansonella perstans* (mf+)		
	Mali	15
	Uganda	5
	Total	20
Other helminth infections		
*Strongyloides stercoralis*		10
*Ascaris lumbricoides*		4
Hookworm spp.		2
	Total	16

* Circulating antigen negative.

### LIPS assay

The LIPS assay detects IgG or IgG4 antibodies to an antigen (Wb123) and was performed exactly as described previously [18] except that an input of 10 million luminometer units (LU) of the enzyme reporter Ruc-Wb123 was used. Briefly, 1 uL of patient sera was diluted 1∶10 in assay buffer (20 mM Tris, pH 7.5, 150 mM NaCl, 5 mM MgCl_2_, 1% Triton X-100) in a 96-well polypropylene microtiter plate (Nunc, Roskilde, Denmark) and was added to 50 µl of 1×10^7^ LU of Ruc-antigen in polypropylene plates. The plate was incubated for 5 minutes at room temperature after which the material was added to 7 µl of a 30% suspension of protein A/G beads in PBS (Pierce Biotechnology, Rockford, IL) in a 96-well filter HTS plate (Millipore, Bedford, MA). After 5 minutes, the filter plate containing the mixture was then applied to a vacuum manifold and washed twice in assay buffer and 8 times with PBS. After the final wash, all plates were processed on a Berthold LB 960 Centro microplate luminometer using a colenterazine substrate mix (Promega, Madison, WI). All data were the average of duplicates corrected for background reactivity (no serum added).

For anti-IgG4 antibody determinations, the same protocol was utilized with anti-IgG4 antibody beads substituted for protein A/G beads. The anti-IgG4 antibody beads were generated by combining 10 mg of an anti-IgG4 monoclonal antibody (clone HP6023, Hybridoma Reagent Laboratories, Baltimore, MD) with Ultralink pre-activated beads (Pierce Biotechnology), as described by the manufacturer. The coupling efficiency was greater than 90%.

### Sequence alignment

The Wb123 amino acid sequence was run against databases for other filariae using the basic alignment search tool (BLAST). This sequence was then to aligned to the related filarial sequences using ClustalW [19] with the default parameters.

### Statistical analysis

Geometric means were used as measurements of central tendency. For determining the cut-off limits receiver operating characteristic (ROC) curves were used. ROC analysis originates from signal detection theory as a model of how well a receiver is able to detect a signal in the presence of noise. Its key feature is the distinction between a true positive rate (sensitivity) and a false positive rate (specificity). The non-parametric Mann-Whitney *U* test was used for comparison of antibody levels in different groups. The Spearman rank correlation was used for correlation analyses. All statistical analyses were performed using GraphPad (v5).

## Results

L3-derived ESTs from *W. bancrofti* (n = 2048), *B. malayi* (n = 5068), *L. loa* (n = 3351) and *O. volvulus* (n = 4168) were each assembled into contigs by dCAS analysis (906 for Wb, 1649 for Bm, 1433 for Ll and 1530 for Ov). From these, 19 potential candidates were identified by virtue of having limited similarity to all publicly available nematode ESTs, to the non-redundant protein database (nr) and to clustered ESTs derived from filarial microfilariae and adult parasites. Each candidate transcript included a start codon and stop codon separated by at least 200 base pairs, indicating a potential open reading frame (ORF). The 19 candidates (and their annotations) are shown in Table S1.

Sixteen of these 19 were successfully cloned in pREN2 and used to transfect COS1 cells. The lysates were used in a standard LIPS format, and only 1, termed Wb123 (Genbank Accession number HQ438580), was shown to be both immunogenic and *W. bancrofti*-specific in preliminary testing.

### Characteristics of Wb123

**Figure 1:**
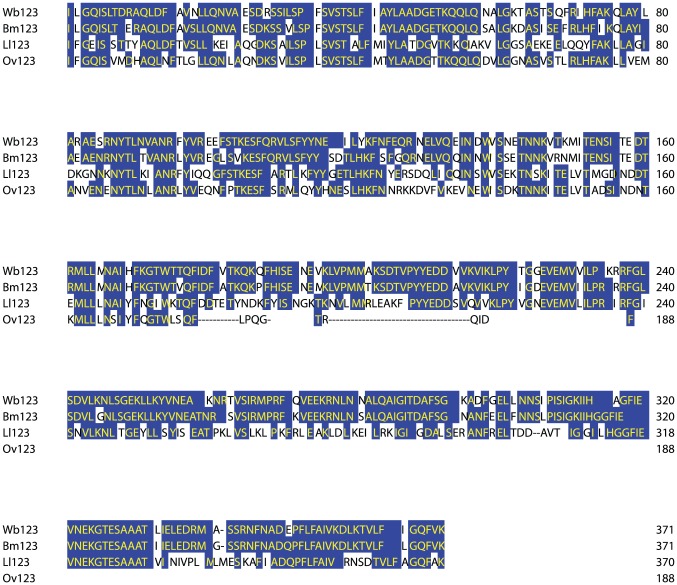
Sequence alignment of Wb123. Sequence alignment of Wb123 with homologous proteins in *Brugia malayi* (Bm123), *Loa loa* (Ll123), and *Onchocerca volvulus* (Ov123). Boxed blue amino acids are those that show 100% conservation with those of Wb123.

Wb123 contains 372 amino acids. The annotation of its sequence suggested a “hypothetical protein,” but it likely belongs to the serpin family of proteins. These are serine protease inhibitors that have been shown to be secreted proteins in filarial species other than Wb, often L3 enriched, and have also been shown to be highly immunogenic in humans. Although there are human serpins, these share little to no homology to Wb123 (data not shown). Amino acid sequence alignment between Wb123 and homologous sequences in Bm (82% identity), Ov (35% identity), and Ll (61% identity) is shown in Figure 1.

### Performance of Wb123 in LIPS-based IgG and IgG4 assays

**Figure 2:**
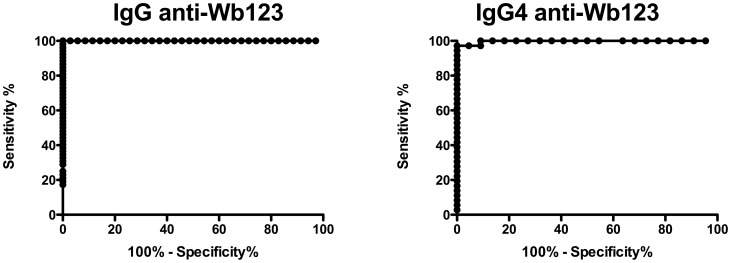
ROC Analysis Wb123 antibody responses. Receiver operating characteristic (ROC) analysis using serum samples from *Wuchereria bancrofti*-infected subjects (n = 43) and normal North American non-travelers (n = 50) for IgG (Left panel) and IgG4 (Right panel) anti-Wb123 antibodies.

To assess the diagnostic utility of Wb123, sera from 52 uninfected normal North Americans were compared to sera from 42 Wb-infected individuals using assays that detected either IgG or IgG4 antibody against Wb123. By ROC curve analysis (Figure 2), a cutoff of 10968 LU was shown to be 100% sensitive and 100% specific in distinguishing the Wb-infected sera from the 52 uninfected control sera in IgG; for IgG4-specific antibodies, a cutoff of 2182 LU was shown to be 97% sensitive and 100% specific using the same sera (Figure 2). Not surprisingly, there was a strong correlation between the IgG and IgG4 anti-Wb123 antibodies (p = 0.0016, r = 0.55).

**Figure 3:**
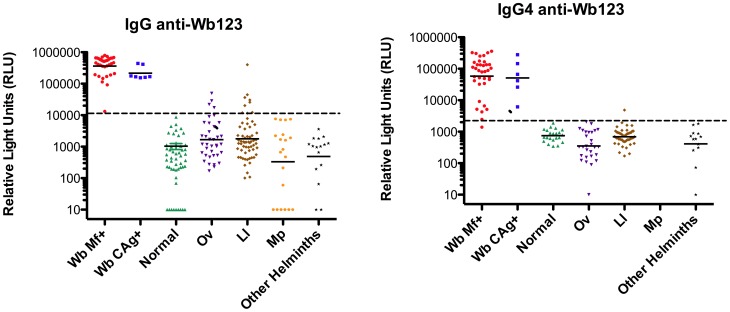
Wb123-specific IgG and IgG4 in *W. bancrofti* and related helminth infections. IgG and IgG4 antibodies to Wb123 distinguish *Wuchereria bancrofti*-infected subjects from those with related filarial and non-filarial helminth infection. IgG (Left panel) or IgG4 (Right panel) antibodies to Wb123 in individual serum samples from those with *Wuchereria bancrofti* (Wb), *Onchocerca volvulus* (Ov), *Loa loa* (Ll), *Mansonella perstans* (Mp), other helminth infections, or no infection (Normal). Each dot represents an individual sample and the horizontal line is the geometric mean (GM). The dashed line represents the cutoff between negative and positive based on ROC analysis.

When panels of sera from well defined patient groups (Table 1) were tested, the Wb123 IgG and IgG4 LIPS assays were both capable of distinguishing Wb infection not only from normal individuals, but also from Ll-, Ov-, *Mansonella perstans* (Mp)- and other non-filarial helminth-infected individuals (Figure 3). The Wb123 IgG assay identified all of the Wb-infected individuals with no cross-reactivity among patients with Mp or other non-filarial helminths (e.g. *Strongyloides stercoralis*, *Ascaris lumbricoides*, hookworm). There was, however, a small degree of IgG cross-reactivity (4/38 samples for Ov and 5/62 samples for Ll). In contrast, the IgG4 Wb123 assay (Figure 3 Right panel) showed a higher degree of specificity (only a single Ll-infected patient being positive) with only a minimal loss in sensitivity (1/42 Wb positive sera below the cutoff).

The performance characteristics of the Wb123 IgG and IgG4 LIPS assays for distinguishing Wb infection from infection with closely related and potentially geographically overlapping filarial infections are shown in Table 2. Sensitivity of IgG-based LIPS assays ranged from 98–100% and specificity ranged from 89–100% with respect to the related Mp, Ll, Ov infections, with correspondingly good positive predictive values (PPV) and negative predictive values (NPV). For the IgG4-based LIPS assays, sensitivity was slightly lower (98%) but specificity higher (99–100%) with equally good PPVs and NPVs.

**Table 2:** Sensitivity and specificity of Wb123 IgG and IgG4 LIPS.

Compared to*	Normals	*L. loa*	*O.volvulus*	*M. perstans*	Other Helminths
**Sensitivity**					
IgG	100	100	100	100	100
IgG4	98	98	98	ND	98
**Specificity**					
IgG	100	89	90	100	100
IgG4	100	99	100	ND	100
**PPV** **					
IgG	100	87	90	100	100
IgG4	100	98	100	ND	100
**NPV** ***					
IgG	100	100	100	100	100
IgG4	96	99	98	ND	95

* Calculations (Sensitivity, Specificity, PPV, NPV) are shown when the positive was defined as sera from proven *Wuchereria bancrofti* infection and the negative was defined as either normal (Normals), *Loa loa*-infected (*L. loa*). *Onchocera volvulus*-infected (O. *volvulus)*, *Mansonella perstans*-infected (*M. perstans*) or infected with soil transmitted helminths (Other Helminths).

ND = not done.

** Positive Predictive Value.

*** Negative Predictive Value.

### Testing in blinded samples

**Figure 4:**
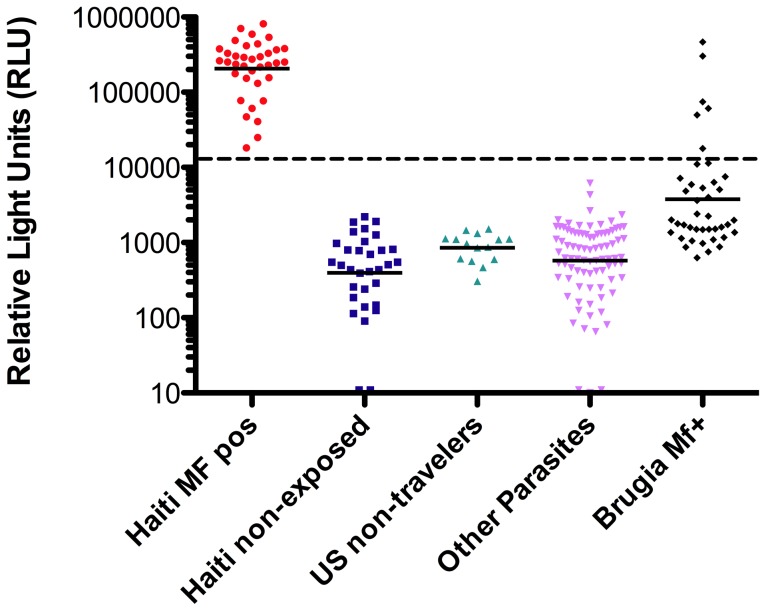
Performance of Wb123-specific IgG LIPS assay in blinded analysis. Blinded analysis of IgG anti-Wb123 antibodies from samples obtained from *Wuchereria bancrofti*-infected patients from Haiti (Haiti MF pos), those from a Wb non-endemic region of Haiti (Haiti non-exposed), US non-travelers, those with other non-filarial parasitic infection, or those with *Brugia malayi* infection. Each dot represents an individual subject and the horizontal line is the GM. The dashed line represents the cutoff between negative and positive based on ROC analysis.

Sera from well-characterized cohorts of patients were next tested in a blinded fashion using the IgG Wb123 LIPS (Figure 4). Wb123 LIPS performed extraordinarily well in distinguishing Wb-infected Haitian individuals from both endemic (Haitian) normal individuals as well as from non-endemic travelers with a variety of proven non-filarial parasitic infections (see Table S2 for details). Moreover, when sera from microfilaria positive (mf+) individuals with *Brugia malayi* were tested, there was relatively little (6/41) IgG reactivity in the Wb123 LIPS despite the relatively high degree of conservation between Wb123 and the Bm123 homologue (see Figure 1).

### Antibodies to Wb123 diminish but do not disappear following definitive treatment

**Figure 5:**
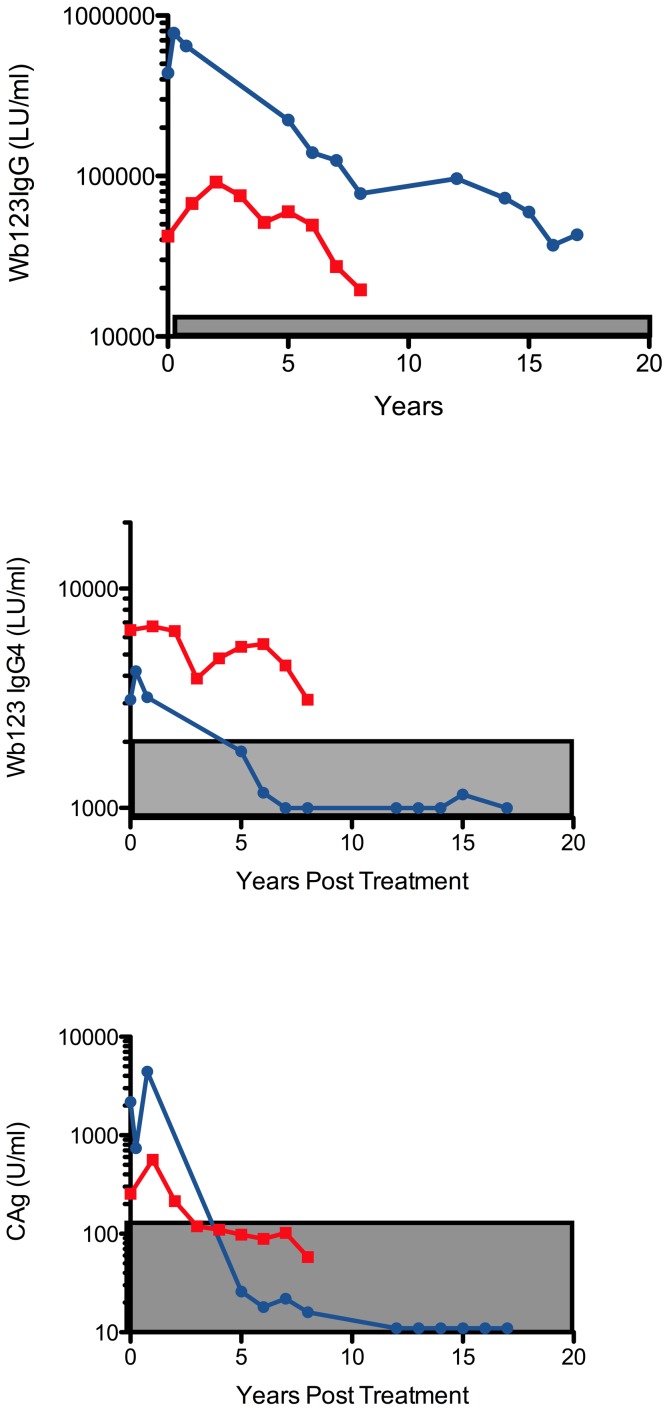
Antibodies to Wb123 fail to normalize following definitive anti-filarial treatment. IgG (Top panel) and IgG4 (Middle panel) anti-Wb123 antibodies in two individual patients followed longitudinally over an 8 (red line) or 17 (blue line) year period following definitive anti-filarial chemotherapy. Circulating antigen levels in the same two subjects is shown (Bottom panel). Gray shaded boxes show the normal range for each assay.

We had the opportunity to examine the decline in antibody following definitive treatment in two individuals with Wb infection who had acquired their infection during early life in Guyana. Each of these individuals was treated (multiple courses of diethylcarbamazine and/or doxycycline) and followed for up to 17 years with neither having returned to a Wb endemic country. As can be seen in Figure 5, Wb123-specific IgG and IgG4 antibodies declined for each individual following treatment, but the magnitude of the decline was neither statistically significant nor did it fall below the cutoff levels for the IgG Wb123 assay. Persistence of detectable anti-Wb123 antibody occurred despite the loss of circulating filarial antigenemia in the two subjects.

## Discussion

The purpose of this study was to identify a Wb- and L3-specific antigen that could potentially be used to monitor changes in transmission of *W. bancrofti* following mass drug administration (MDA) in areas of the world (e.g. Africa, South and Central America) where the geographical overlap of Ov, Ll, and Mp infections has caused significant cross reactions to antigens currently being used in antibody-based assays. Wb123, a serpin-like Wb-encoded protein, appears to be a good candidate for immunoassays based on the extraordinarily good sensitivity and specificity observed with this antigen in the LIPS format. Not only can Wb123-based assays distinguish Wb-infected individuals from uninfected individuals, but they can also be used to distinguish Wb infection from infections with the closely related filarial parasites, Bm, Ll, Ov, and Mp. Furthermore, the Wb123 LIPS assay is a convenient diagnostic for field use as it provides a rapid, high throughput format that requires minimal serum volume (1 uL per test) and can be used with serum, whole blood, filter spotted material, or saliva (data not shown).

Unlike PCR, microscopy, and antigen detection assays, antibody detection assays in LF cannot definitively identify active infection, nor can they distinguish between past and current infection. However, antibody tests can be used for surveillance activities to estimate infection prevalence rates [12], [20], [21]. Anti-Wb123 antibodies develop months to a year before the appearance of blood microfilariae (see accompanying article by Hamlin *et al*). Thus, the rapid-format Wb123 LIPS can provide early identification of new infections and, by inference, ongoing transmission. The Wb123 LIPS test could be used for rapid screening of populations and thereby provide a means by which control programs could be more accurately monitored.

One of the greatest needs of the GPELF is for a highly sensitive and specific surveillance tool to monitor, in sentinel populations [22], [23], exposure to filarial infection in regions that have (or are about to have) ceased annual MDA [22], [24]. With MDA programs nearing or having reached completion in 13 countries, the availability of a surveillance tool like Wb123 antibody detection becomes highly valuable. Though highly specific diagnostic tests for detecting active infection, such as the ICT card [25] and the Og4C3 ELISA, are well established [23], [26]–[30], a persistent challenge has been to devise an assay that is both sensitive and specific enough to be used in *W. bancrofti*-endemic countries that are also endemic for other filarial infections, particularly Ll, Ov, and Mp. Although there have been developed many sensitive antibody assays [12]–[15], [20], [22], [23], [26], [29], [31], almost all ELISA-based, none has had the specificity sufficient to meet the current needs for monitoring in Africa and/or the Americas. The Wb123 LIPS assay was developed to meet this need and has the ability to detect exposure to Wb infective L3s with little or no cross reactivity in the face of concomitant non-Wb filarial infections.

This study also demonstrates that Wb123 LIPS can detect *Wb* infection by antibody profiling with high diagnostic sensitivity and specificity. The high-throughput, rapid (and partially automated) formats used here make these approaches highly feasible for screening large numbers of sera samples and/or for rapid large scale field-testing. Similar to our previous published results with Ss [18], Ll [32] and Ov [33], the Wb123 LIPS showed a large dynamic range in Wb-positive sera and relatively low background binding values in uninfected control sera.

Because levels of antibody to Wb123 slowly decrease after definitive treatment (see Fig. 5), the Wb123 assays are likely unhelpful in populations exposed to *W. bancrofti* before the implementation of MDA programs. Therefore, the target population for this field (or region)-applicable Wb123 LIPS (and other immunoassay formats such as ELISA and/or Luminex using IgG4 detection strategies) would be school age children (6–7 year olds), a population that would provide an efficient target for the detection of recent infection and (by inference) recent or ongoing transmission. Prospective testing of this concept is underway in Mali and American Samoa as are efforts to re-format the Wb123 LIPS assay into a true point of care diagnostic.

## Supporting Information

Table S1 **Annotated Excel table ouput from dCAS of 19 antigens used as potential targets for** ***Wuchereria bancrofti*** **and/or** ***Brugia malayi*** **-specific antibody assays.**
(XLSX)

Table S2 **Composition of serum samples used for blinded analyses of IgG anti-Wb123 LIPS assay.**
(DOCX)
