# Supplementary material for: Identification of Wb123 as an Early and Specific Marker of Wuchereria bancrofti Infection
Source: PLoS Negl Trop Dis. 2012 Dec 6;6(12):e1930. doi: 10.1371/journal.pntd.0001930 (PMC3516582; doi:10.1371/journal.pntd.0001930)
Supplement: Table S2 — Composition of serum samples used for blinded analyses of IgG anti-Wb123 LIPS assay. (DOCX) [file pntd.0001930.s002.docx]

Supplemental Table 2 - Composition of serum panels run in blinded analyses for Wb123 IgG antibodies

| Group | Country where applicable | Number |
| --- | --- | --- |
|  |  |  |
| *Wuchereria bancrofti* |  |  |
|  | Haiti (mf+) | 37 |
|  |  |  |
| *Brugia malayi* |  |  |
|  | India (mf+) | 34 |
|  | Indonesia (mf+) | 7 |
|  | Total | 41 |
|  |  |  |
| Uninfected (non-exposed) | Haiti | 32 |
|  |  |  |
| US non-travelers | United States | 15 |
|  |  |  |
| **Other Infections** |  |  |
| Zoonotic Filariasis |  | 6 |
| Toxoplasma |  | 1 |
| Hookworm |  | 17 |
| Schistosoma spp. |  | 7 |
| Tuberculosis |  | 1 |
| Baylisascaris |  | 1 |
| *H. nana* |  | 4 |
| Ascaris |  | 5 |
| Cysticercosis |  | 5 |
| Taenia spp. |  | 5 |
| Echinococcus |  | 6 |
| Entamoeba spp. |  | 3 |
| E*. nana* |  | 1 |
| Fasciola |  | 3 |
| Paragonimus |  | 6 |
| Toxocara |  | 5 |
| Trichinella |  | 3 |
| Trichuris |  | 7 |
|  | Total | 86 |
